# Identification of an Endophytic Antifungal Bacterial Strain Isolated from the Rubber Tree and Its Application in the Biological Control of Banana Fusarium Wilt

**DOI:** 10.1371/journal.pone.0131974

**Published:** 2015-07-02

**Authors:** Deguan Tan, Lili Fu, Bingyin Han, Xuepiao Sun, Peng Zheng, Jiaming Zhang

**Affiliations:** 1 MOA Key Laboratory of Biology and Genetic Resources for Tropical Crops, Institute of Tropical Bioscience and Biotechnology, CATAS, Haikou, Hainan Province, 571101, China; 2 Lijiang Teachers College, Lijiang, Yunnan Province, 674110, China; National Key Laboratory of Crop Genetic Improvement, CHINA

## Abstract

Banana Fusarium wilt (also known as Panama disease) is one of the most disastrous plant diseases. Effective control methods are still under exploring. The endophytic bacterial strain ITBB B5-1 was isolated from the rubber tree, and identified as *Serratia marcescens* by morphological, biochemical, and phylogenetic analyses. This strain exhibited a high potential for biological control against the banana Fusarium disease. Visual agar plate assay showed that ITBB B5-1 restricted the mycelial growth of the pathogenic fungus *Fusarium oxysporum f*. *sp*. *cubense* race 4 (FOC4). Microscopic observation revealed that the cell wall of the FOC4 mycelium close to the co-cultured bacterium was partially decomposed, and the conidial formation was prohibited. The inhibition ratio of the culture fluid of ITBB B5-1 against the pathogenic fungus was 95.4% as estimated by tip culture assay. Chitinase and glucanase activity was detected in the culture fluid, and the highest activity was obtained at Day 2 and Day 3 of incubation for chitinase and glucanase, respectively. The filtrated cell-free culture fluid degraded the cell wall of FOC4 mycelium. These results indicated that chitinase and glucanase were involved in the antifungal mechanism of ITBB B5-1. The potted banana plants that were inoculated with ITBB B5-1 before infection with FOC4 showed 78.7% reduction in the disease severity index in the green house experiments. In the field trials, ITBB B5-1 showed a control effect of approximately 70.0% against the disease. Therefore, the endophytic bacterial strain ITBB B5-1 could be applied in the biological control of banana Fusarium wilt.

## Introduction

Banana is among the most important food and fruit crops in many developing countries [1]. However, diseases and pests became severe problems when certain genotypes were cultivated as monocultures [2], and limited the increase of banana production in the last few decades [2]. Fusarium wilt (also known as Panama disease) is one of the most notorious and destructive diseases in banana [3]. It has been reported in all banana-producing countries, including Asia, Central and South America, Africa, and Australia [4].

The pathogen of banana Fusarium wilt was identified as a soil-borne hyphomycete, *Fusarium oxysporum* formae specialis *cubense* (FOC) [4–6], and was classified into four physiological races based on virulence to host cultivars in the field [7, 8], including FOC1, FOC2, FOC3, and FOC4. FOC1 infects the cultivars Gros Michel (AAA group), Silk (AAB group), and Pisang Awak (ABB group); FOC2 infects Bluggoe (ABB group) and its close relatives; FOC3 infects *Heliconia spp*.; and FOC4 infects Cavendish cultivars (AAA group) and all the cultivars susceptible to FOC1 and FOC2 [8].

Before 1960, the cultivar Gros Michel was dominant and supplied almost all the export trade. However, this cultivar was susceptible to FOC1, and the accident utilization of infected rhizomes or suckers to establish new plantations caused the widespread of the disease, and the world’s banana industry was almost destroyed [3]. As a result, Cavendish cultivars that were resistant to FOC1 were cultivated to replace Gros Michel worldwide [9]. These cultivars remain to be the well-performed clones in the western tropics. However, they are not resistant to FOC4 identified in the eastern tropics, and caused serious crop losses in the Cavendish banana plantations in Asia, Australia, and Africa [10]. For example, the banana plantations in China, including Hainan, Guangdong, Guangxi, Yunnan, and Fujian Provinces have encountered an estimated average disease incidence of 20 to 40% [11], with the highest rate of 90% [12–14]. Although FOC4 exists only in the eastern tropics, there is also concern over the spreading of FOC4 to the western tropics, since banana production is predicted to be destroyed completely in that region if FOC4 invaded [15].

To control the disease, fungicides, biocontrol agents, and resistant cultivars have been investigated [2, 10, 13, 16–23]. Molecular breeding has also been used in the developing of resistant varieties [24, 25]. Significant mycelial growth or disease control was achieved in some of these studies in the laboratory and/or in the green house, but the field demonstrations were mostly not satisfactory.

We have isolated an endophytic bacterial strain from the rubber tree, and identified it as a *Serratia marcescens* strain with a special intracellular secretion apparatus and antifungal activity. This strain had a high potential in the biological control of banana Fusarium wilt.

## Materials and Methods

### Isolation of the bacterial strain ITBB B5-1

The bacterial strain *Serratia marcescens* ITBB B5-1 was isolated from sterilized young branches of the rubber tree (*Hevea brasiliensis* clone Reyan 7-33-97) grown in the green house at the Institute of Tropical Bioscience and Biotechnology, Chinese Academy of Tropical Agricultural Sciences (CATAS), Haikou, China. Young branches were removed from healthy rubber trees, segmented to approximately 4 cm, and washed with distilled water. The segments were then rinsed in 75% ethanol for 30 s, and sterilized in 0.2% HgCl_2_ for 8 min, followed by washing with sterilized distilled water four times. The segments were cut into thin slices (1–2 mm) and incubated on agar-solidified Luria-Bertani (LB) medium[26] in the dark at 28°C for 4–10 days. The ITBB B5-1 strain that produced a red pigment was isolated and maintained on LB plates or stored at -80°C. This strain was also deposited at the China General Microbiological Culture Collection Center located in Beijing, China under accession number CGMCC7416.

### Morphological characterization

ITBB B5-1 bacterial cells were mounted on glass slides, with or without staining by Gram-stain reagents, and examined using a light microscope (Olympus BH2, Japan). Photographs were taken under an oil immersion objective lens (100X).

For scanning electron microscopy, cells in late exponential growth were collected from suspension cultures in LB broth by centrifugation (5000 rpm, 5 min), washed twice with 0.01M phosphate buffered saline (PBS, 0.228 g NaH_2_PO_4_, 1.15 g Na_2_HPO_4_ in 1 L ddH_2_O) and fixed in 0.5% glutaraldehyde and 1% formaldehyde. The cells were dehydrated through a series of acetone solutions, spreaded over glass coverslips, critical point dried, and then dressed with gold. The samples were then observed under a JSM-35C scanning electron microscope (JEOL Ltd., Japan).

For transmission electron microscopy, cells were collected from LB plates or suspension cultures, fixed with 2% glutaraldehyde and 1% formaldehyde dissolved in 50 mM Tris/HCl buffer (pH 7.4) at 4°C, and harvested by centrifugation at 5000 rpm for 5 min. The cells were washed in 50 mM Na-cacodylate buffer (pH 7.0) and resuspended in 1% osmium tetroxide (aqueous solution) overnight at 4°C, followed by dehydrating through an acetone series. After embedment in Spurr’s resin, ultrathin sections were cut with a diamond knife. The slides were mounted on formvar/carbon-coated slots, sequentially stained with uranyl acetate and lead citrate, and finally observed under a JEOL 1010 transmission electron microscope (JEOL Ltd., Japan).

### DNA extraction and amplification of 16S rDNA

The ITBB B5-1 strain was cultured in LB broth overnight with shaking at 28°C and harvested by centrifugation. Genomic DNA was extracted using the Universal Genomic DNA Extraction Kit (Sangon, Shanghai, China), according to the manufacturer’s instruction. 16S rDNA was amplified using the forward and reverse primers 5′-AGA GTT TGA TCC TGG CTC AG-3′ and 5′-AAG GAG GTG ATC CAG CCG CA-3′, respectively [27]. The fragment was sequenced at Shanghai Sangon Biological Engineering Technology & Services Co., Ltd. The sequence was analyzed using MacVector software (Oxford Molecular, Oxford, UK), and was registered in the GenBank database (JN896750). BLAST search using the 16S rDNA sequence as a query was performed against the GenBank database.

### Phylogenetic analysis

For phylogenetic analysis, 16S rDNAs of the related bacterial strains were obtained from GenBank database and aligned using ClustalX 3.0 [28]. The primer regions were removed from the sequences. The alignment results were exported to MEGA4 [29]. Phylogenetic trees were generated using the Neighbor-Joining (NJ), Minimum Evolution (ME), and Maximum Parsimony (MP) methods with 1000 bootstrap replicates. Only the Minimum Evolution Tree is provided. The evolutionary history was inferred using the ME method [30]. The optimal tree with the sum of branch length = 0.06731820 is shownin this paper. All positions containing gaps and missing data were eliminated from the dataset (complete deletion option). There were a total of 1293 positions in the final dataset. The trees were rooted with 16S rDNA sequences from members of the Enterobacteriaceae family, including *Klebsiella planticola* strain DR3 (X93216), and two other strains.

### The plant pathogenic fungal strain and spore preparation

The pathogenic fungal strain *Fusarium oxysporum f*. *sp*. *cubense* race 4 (FOC4) that caused severe diseases in banana was provided by Dr. Junsheng Huang, Institute of Plant and Environment Protection, Chinese Academy of Tropical Agricultural Sciences (CATAS). The strain was maintained on potato dextrose agar (PDA) medium at 28°C. The FOC4 conidial suspension was prepared by incubating FOC4 in PDA broth on a shaker at 200 rpm, 28°C for 5 days, followed by filtration with two layers of gauze to remove the mycelia. The conidial concentration was adjusted to 1×10^6^ conidia per ml, and was stored at 4°C till utilization.

### Visual agar plate assay of the antifungal effect of ITBB B5-1 against FOC4

The ITBB B5-1 strain suspension and the suspensions of the control strains *Escherichia coli* DH5α and *Agrobacterium tumefaciens* EHA105 were prepared by incubating the strains in LB broth with shaking at 250 rpm, 25°C for 2 days, followed by dilution of the bacteria to 10^8^ cfu per ml with distilled water, and storage at 4°C.

The visual agar plate assay [31] was used to test the inhibition effect of ITBB B5-1 on the growth of the pathogenic strain of FOC4. The FOC4 conidial suspension prepared as described above was inoculated in a line at the center of LB plates. Then an aliquot of the bacterial suspension of ITBB B5-1 was inoculated in the right and left lines 2 cm away from the central line; LB medium and/or the suspensions of *E*. *coli* DH5α and *A*. *tumefaciens* EHA105 were used at the position of ITBB B5-1 as controls. The plates were incubated at 26°C. The width of the mycelial line was measured every day, and the significance of the inhibition effect was tested with one-way ANOVA method at 1% confidence level.

For microscopy of the FOC4 mycelia at the frontier of the fungal line in the visual agar plate assay, the frontier mycelia were mounted on glass slides, and stained with an Ehrlich hematoxylin and eosin staining reagent (Leagene, Beijing) for 20 min, washed with deionized water and 1% ammonium solution for 30 sec each, and covered with glass slips. The samples were observed and photographed under a light microscope (Axioskop 40, Zeiss, Germany).

To confirm the lytic activity of culture medium of ITBB B5-1, the FOC4 mycelia were mounted on glass slides, and treated with filtrate of the culture fluid of the strain ITBB B5-1 for 30 minutes, and observed under microscope.

### Quantitative assay of the antifungal function against FOC4

The ITBB B5-1 strain was cultured in LB broth on a shaker at 250 rpm and 25°C for 2 days, and centrifuged at 4, 200 g for 20 min to remove the bacteria. The supernatant was filtrated with 0.22 μm filter units (Millipore, Bedford, USA) to remove remaining bacteria. The filtration was stored at 4°C. The inhibition effect of the filtrate against FOC4 was quantified by a tip culture method [32]. Five ml pipette tips were used as culture vessels by sealing the tip with paraffin. 100 μl of the above filtrate was added to 700 μl PDA broth in the pipette tips, using 100 μl LB broth as control. Each tip was inoculated with 10 μl FOC4 conidial suspension prepared as described above, and incubated at 28°C for 6 days. The paraffin was removed from the tip, and the mycelia of FOC4 were collected by gentle centrifugation at 96 g for 5 min. The mycelia were weighed using a balance of 0.1 mg accuracy (Mettler Toledo, AL204). The antifungal ratio was calculated by the equation: (Weight of control—weight of treatment) / weight of control × 100%. The experiment was replicated three times, and the significance of the inhibition was tested with one-way ANOVA at 1% confidence level.

### Assay of chitinase and glucanase activity of ITBB B5-1

A single colony of the ITBB B5-1 strain was incubated in 3 ml LB broth at 25°C for 24 h with shaking at 250 rpm. Five μl of the activated bacterial fluid was inoculated into each 10 ml culture tube containing 3 ml of LB broth, and 30 tubes were used. The tubes were incubated at 250 rpm and 25°C. Samples were collected at 12 h intervals, 3 tubes each time. The samples were centrifuged at 4, 200 g for 10 min. The supernatant was collected and filtered through 0.22 μm filter units (Millipore, Bedford, USA) to remove remaining bacteria. Chitinase activity of the filtrate was measured as described previously [33]. One unit of chitinase activity was defined as the amount of enzyme that catalyzed the release of 1 μmol of N-acetylglucosamine per hour at 50°C. β-1, 3-glucanase activity was determined as described previously [34] with a few modifications. The reaction mixture contained 50 μl filtrate, 250 μl laminarin (Sigma) (2 mg·ml^-1^ laminarin in 50 mM sodium acetate buffer, pH 5.0), and 200 μl 50 mM sodium acetate (pH 5.0). The reaction was incubated at 38°C for 3 h, and then 500 μl copper reagent was added in the reaction. The reaction was boiled for 10 min and quickly cooled down to room temperature. 1 ml of arsenomolybdate solution was then added into the reaction mixture for color development. The absorbance was measured at 500 nm with a spectrophotometer (Bio-tech, USA). One unit of β-1, 3-glucanase activity was defined as the amount of enzyme that catalyzed the release of 1 μmol of glucose per hour.

### Green house test of the biocontrol function of ITBB B5-1 against FOC4

One-month-old nursery banana plants (variety Williams, *Musa* AAA Cavendish subgroup) were bought from the Tissue Culture Factory of the CATAS. The plants were grown in plastic pots of 15 cm in diameter and 10 cm in depth, and were claimed to be disease free. The biological control function of the ITBB B5-1 strain against FOC4 was carried out in the green house of the Institute of Tropical Bioscience and Biotechnology, CATAS.

Before infection with FOC4, the plants were treated with 100 ml ITBB B5-1 suspension prepared as described above, and repeated once 3 days later. The plants treated with only LB broth diluted to the same ratio as the bacterial suspension were used as a control (CK1). Fifteen days after inoculation with ITBB B5-1, each pot was watered with 100 ml Fusarium conidial suspension (1×10^6^ spores / ml) prepared as described above. The plants that were watered with only the PDA medium were used as another control (CK2). The treatments and controls were done in triplicate consisting of 10 plants per replicate. Two months later, the disease symptoms were recorded based on the five grade scale from 0 to 4 as described previously [35]: 0-corm completely clean, plant healthy; 1-isolated points of discoloration in vascular tissue; 2-discoloration up to 1/2 of vascular tissue, slight chlorosis in leaves; 3-discoloration greater than 1/2 of vascular tissue, moderate or severe chlorosis in leaves; 4-total discoloration of vascular tissue, plant dead. The disease severity index was calculated using the formula described by Huang et al.[13]. Disease severity index = [∑(Class × Number of that class) / (4 × Total number of assessed plants)] × 100. Based on disease severity index, the control effect was calculated as follows: Control effect (%) = [(Disease severity index of control—Disease severity index of treatment)/ Disease severity index of control] × 100. The significance was analyzed with one-way ANOVA test at 5% confidence level.

### Field trials

To further confirm the biological control function, field trials were performed in Chengxi, Haikou City. One month-old nursery plants (variety Williams, *Musa* AAA Cavendish subgroup) were treated with 100 ml ITBB B5-1 suspension prepared as described above, and repeated once 3 days later. Plants treated with only LB medium diluted as the bacterial suspension were used as control. Fifteen days after inoculation of ITBB B5-1, each pot was watered with 100 ml Fusarium spore suspension prepared as described above. Fifteen days later, the plants were transplanted in the field that was free of the Fusarium disease at 2 m × 2 m intervals. Each treatment and control contained 3 blocks of 10 plants, and the blocks were arranged at intervals. The restriction effect was surveyed 6 months later. The disease severity index and control effect were calculated as described above. The significance was analyzed with one-way ANOVA at 5% confidence level.

## Results

### Light and Electron Microscopy characterization of the endophytic bacterial strain ITBB B5-1

**Fig 1:**
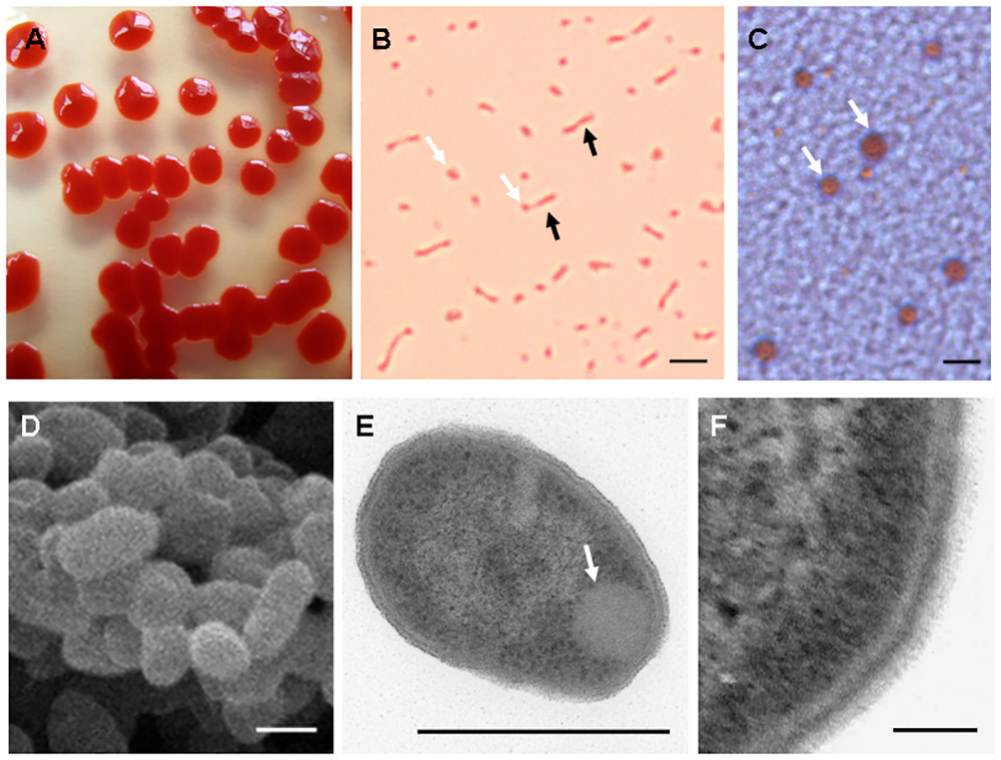
Light and electron microscopy of *S*. *marcescens* strain ITBB B5-1. A, Morphology of the clones on LB plates; B, light microscopy image of the bacterial cells (black arrows) and the extracellular vesicles (white arrows) shown by their original red color, scale bar = 0.5 μm; C, Light microscopy of the pelleted bacterial cells, white arrows indicate the fused vesicles in natural color, scale bar = 2 μm; D, Scanning electron microscopy, scale bar = 0.5 μm; E, Transmission electron microscopy, white arrow indicates the intracellular structure, scale bar = 0.5 μm; F, The cell wall structure under transmission electron microscope, scale bar = 0.1 μm.

The bacterial strain ITBB B5-1 was isolated from sterilized stem segments of the rubber tree. Its clones on LB medium were round, red, and opaque with a wet, convex, and smooth surface (Fig 1A). Conventional physiological and biochemical examinations revealed that the cells were Gram-negative, motile, oxidase-positive, catalase-positive, and methyl red-negative. Light microscopy showed that the cells were red and rod-shaped, with secreted red vesicles (Fig 1B). Observation of the pelleted cells indicated that the red vesicles fused with each other and formed much larger 0.5–2 μm vesicles (Fig 1C). According to these features, the ITBB B5-1 strain was initially identified as *Serratia marcescens*. The pigment was assumed to be prodigiosin produced by some *S*. *marcescens* strains [36, 37].

Scanning electron microscopy showed that the cells were coccobacillus, and had numerous short and thin flagella surrounding the cells (Fig 1D). Transmission electron microscopy revealed that the cells had a triple-layer cell wall, in which the inner and outer layers had low electron density and the central layer had high electron density. Additionally, the surface fimbriae, which was important to pathogenic strains for host infection [38], was absent (Fig 1E and 1F). This feature was different from similar observations of some human pathogenic and environmental strains of *S*. *marcescens* [38–41].

### Sequence analysis and phylogenetic classification of the ITBB B5-1 strain

**Fig 2:**
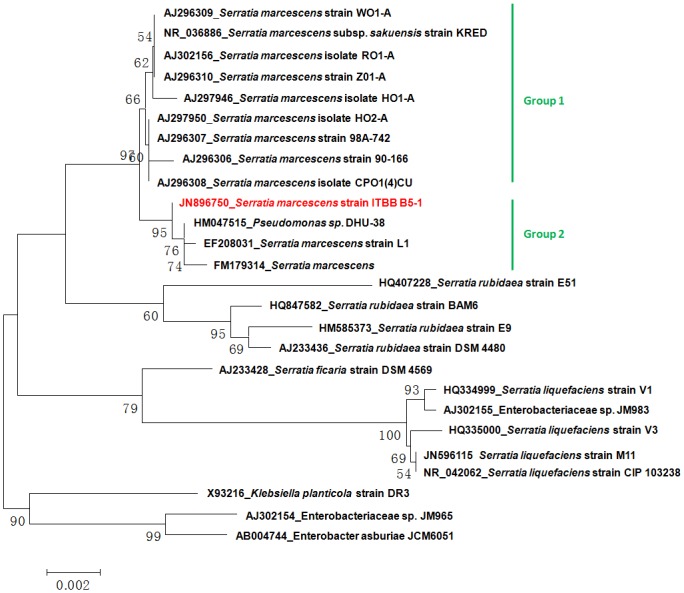
Phylogenetic classification of the ITBB B5-1 strain based on 16S rDNA sequences.

The amplified 16S rDNA sequence of ITBB B5-1 was 1534 bp, with a GC content of 54.51%. Blast searches resulted in the highest similarity with *S*. *marcescens* strain EF208031, with an identity of 99%. Phylogenetic analyses indicated that the four *Serratia* species were clearly separated. ITBB B5-1 was clustered within the *S*. *marcescens* clade with bootstrap supports of 93%, 95%, and 60% when NJ, ME, and MP methods were used, respectively (Fig 2; the NJ and MP trees are not shown). Moreover, the ITBB B5-1 strain was classified in subgroup 2 of *S*. *marcescens*, together with the environmental strains *Pseudomonas sp*. DHU-38 (HM047515), *S*. *marcescens* strain L1 (EF208031), and *S*. *marcescens* strain Pakistan:Lahore (FM179314). Thus, ITBB B5-1 conformed to *S*. *marcescens* strains.

### Antifungal effect of ITBB B5-1 against FOC4

**Fig 3:**
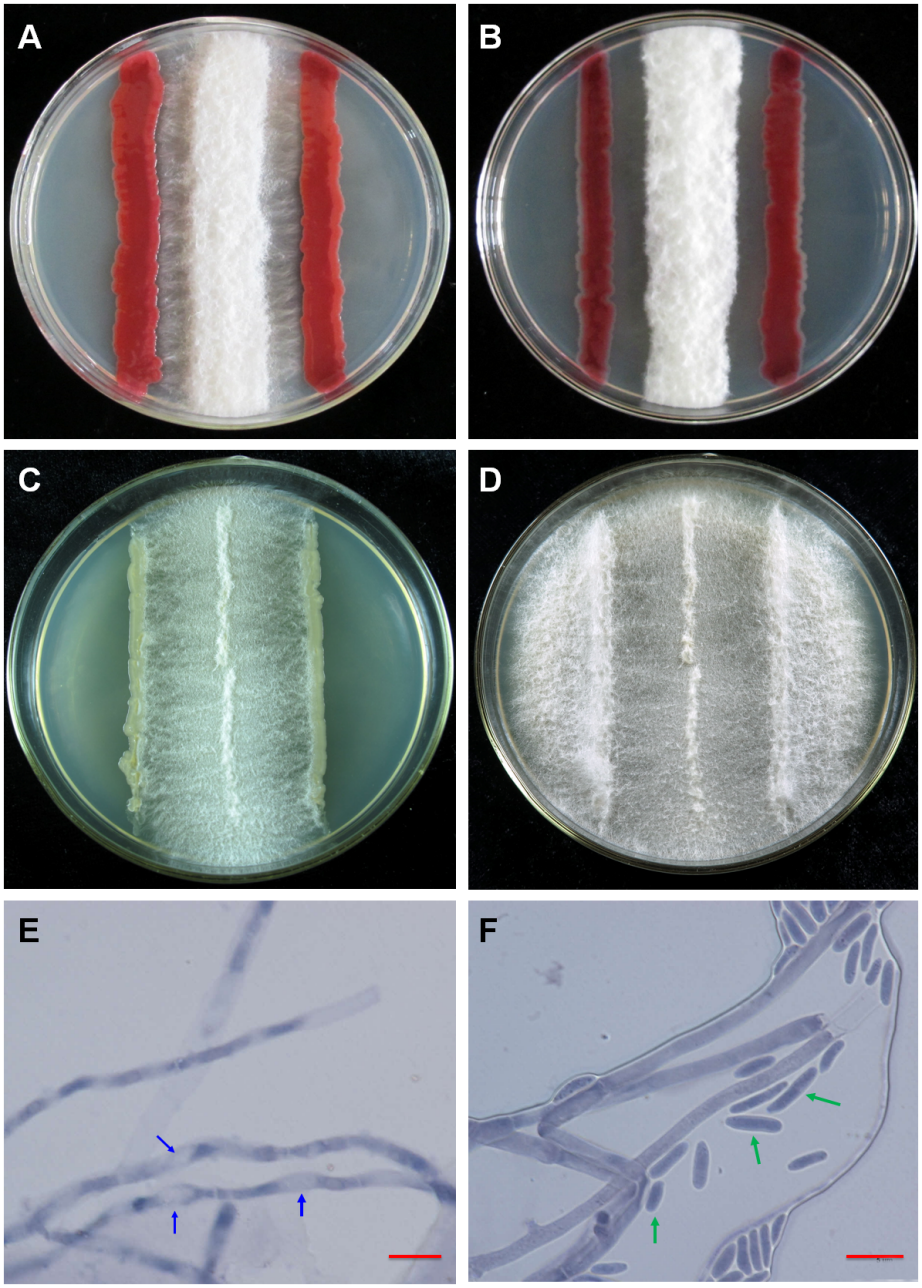
Restriction effect of ITBB B5-1 on the mycelial growth of FOC4. A-B, Mycelia of FOC4 co-cultured with ITBB B5-1 photographed at Day 4 (A) and Day 8 (B); C-D, Mycelia of FOC4 co-cultured with *A*. *tumerfaciens* EHA105 photographed at Day 4 (C) and Day 8 (D); E-F, Microscopic observation of FOC4 mycelia grown with ITBB B5-1 (E) or EHA105 as control (F). Blue arrows indicate the decomposed cell walls in the mycelia; green arrows indicate the conidia; scale bars represent 10 μm.

The antifungal activity of the ITBB B5-1 strain against the pathogenic fungus of banana Fusarium wilt FOC4 was tested with visual agar plate assay (Fig 3). The restriction effect was not significant when the FOC4 fungal line was far away from the bacterial line during the first two days of incubation (Table 1). The inhibition effect became significant at Day 3, when the fungal line grew closer to the bacterial line. The width of the fungal line was only 3.07 cm at Day 4 when co-cultured with ITBB B5-1 (Fig 3A; Table 1). The growth of the fungal line was restricted between the two ITBB B5-1 lines in the following days, and the mycelial frontier that was close to the ITBB B5-1 line began to collapse at Day 7 (Fig 3B). In contrast, the FOC4 fungal lines in the control plates were 4.10, 4.00, and 3.97 cm in width at Day 4 when LB broth, *E*. *coli* DH5α, and *A*. *tumerfaciens* EHA105 were used as controls, respectively (Table 1). The growth of the fungal lines was not significantly affected by *E*. *coli* and *A*. *tumerfaciens* (Table 1, Fig 3C), and the FOC4 mycelia climbed over the bacterial lines of *E*. *coli* and *A*. *tumerfaciens* and grew to almost a full plate at Day 8 (Fig 3D). Microscopic observation indicated that the cell wall of the frontier mycelia close to the ITBB B5-1 line was partially decomposed, leaving inflated and light-stained spots in the mycelia, and the conidial formation was inhibited (Fig 3E), while the mycelia in the control plates were uniformly stained and the conidial formation was not affected (Fig 3F).

**Table 1:** The width of the FOC4 mycelial lines co-cultured with ITBB B5-1 and control strains (cm).

Days	2	3	4	5	6	7	8
CK-LB	1.63±0.15a	2.97±0.06a	4.10±0.26a	5.10±0.53a	5.87±0.51a	6.60±0.62a	7.20±0.70a
CK-DH5α	1.67±0.06a	2.86±0.12a	4.00±0.10a	5.03±0.15a	5.90±0.17a	6.53±0.31a	7.63±0.21a
CK-EH105	1.60±0.10a	2.83±0.06a	3.97±0.15a	4.87±0.12a	5.77±0.12a	6.73±0.15a	7.70±0.26a
ITBB B5-1	1.53±0.15a	2.47±0.06b	3.07±0.12b	3.23±0.15b	3.27±0.12b	-	-

Note: “-” not measured when there was no further growth. The inhibition effect of ITBB B5-1 against the growth of FOC4 was significant from Day 3 as tested with one-way ANOVA method at 1% confidence level, while the growth of FOC4 was not affected by *E*. *coli* DH5α (CK-DH5α) and *A*. *tumerfaciences* EHA105 (CK-EH105). Different letters indicate significant difference.

**Fig 4:**
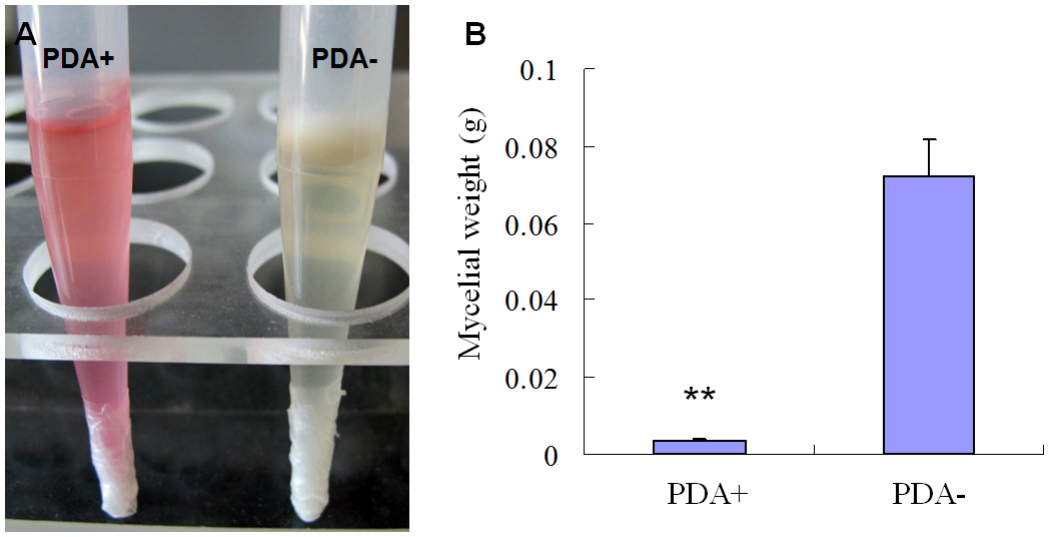
Quantitative assay of the inhibition effect of the culture fluid of ITBB B5-1 on mycelial growth of FOC4. A, tip culture of FOC4 in PDA broth with addition of filtrated medium of ITBB B5-1 (PDA+), or with only addition of LB medium as control (PDA-); B, mycelial weight of FOC4 grown in PDA medium with (PDA+) or without (PDA-) ITBB B5-1 culture fluid.

The inhibition function of the culture fluid of ITBB B5-1 was quantified with the tip-culture method (Fig 4A). There was almost no growth for the FOC4 mycelia in the tip containing the cell-free supernatant of the culture medium of the ITBB B5-1 strain, with an average mycelial fresh weight of only 3.3±0.58 mg. The average fresh weight of the mycelia in the control tips was 72.0±9.5 mg. The inhibition ratio was 95.4%, and statistically significant by ANOVA test (Fig 4B).

### Chitinase and glucanase activities of the fermentative fluid of ITBB B5-1

**Fig 5:**
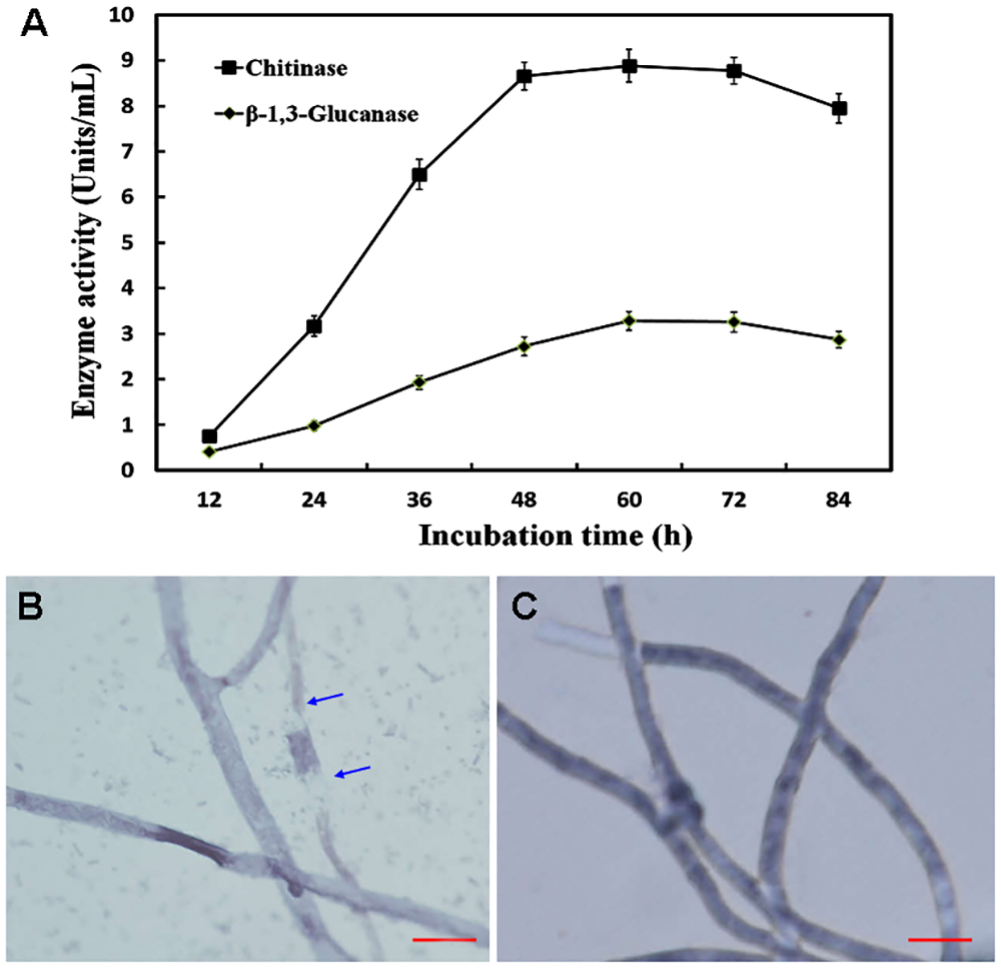
Chitinase and glucanase activity of ITBB B5-1. A, Temporal dependent variation of chitinase and β-1, 3 glucanase activity in the LB broth of ITBB B5-1; B, Mycelia of FOC4 treated with the cell-free culture medium of ITBB B5-1 after 2 days of incubation; blue arrows indicate the degraded mycelia; C, Mycelia of FOC4 treated with only LB broth. Scale bars represent 10 μm.

Chitinase activity of ITBB B5-1 fermentative fluid increased steadily in the initial 48 h of incubation (Fig 5A), and kept a high and stable level of approximately 9 units per ml of the fluid from 48 h to 72 h. The activity then declined slightly at 84 h. β-1, 3-glucanase activity of the fermentative fluid was lower than that of chitinase at all tested time points. However, the temporal dependent pattern of the activity was similar (Fig 5A), which increased in the initial 60 h, and declined at 84 h. The highest glucanase activity was approximately 3 units per ml of the fluid. These results showed that ITBB B5-1 secreted extracellular lytic enzymes chitinase and β-1, 3-glucanase, and could decompose the pathogenic fungi with chitin and β-1, 3-glucan as cell wall components.

Microscopy observation showed that the FOC4 mycelia treated with filtrated fermentative fluid of ITBB B5-1 was partially degraded (Fig 5B), while the control mycelia treated with only LB broth remained intact (Fig 5C). This result indicated that the lytic enzymes secreted by ITBB B5-1 contributed to its antifungal mechanism.

### Inhibitory effect of ITBB B5-1 against banana Fussarium disease in the green house

The potted banana plants in the green house were treated with the ITBB B5-1 strain and infected with FOC4 to test whether ITBB B5-1 could protect the plants against the Fusarium wilt. The plants that were inoculated with ITBB B5-1 before infection with FOC4 had a lower disease severity index than the control plants CK1 that were only treated with LB medium before infection with FOC4. CK1 had a disease severity index of 59.2, and grew significantly weaker with smaller amounts of leaves than the plants protected by ITBB B5-1 (Table 2). The protected plants had similar number of leaves compared to the control plants CK2 that were free of FOC4 (Table 2). The control effect of ITBB B5-1 against Fusarium wilt in the green house experiments was 78.7%.

**Table 2:** Protection effect of the ITBB B5-1 strain on banana plants against Fusarium wilt in the green house.

	Number of leaves (mean±SD)	Disease severity index (mean±SD)	Control effect (%)
T	5.8±0.6 a*	12.5±2.5 b	78.7±5.3
CK1	4.3±0.3 b	59.2±5.2 c	N/A
CK2	6.3±0.7 a	0 a	N/A

Note:

“*” Different letters indicate significant difference within each column using one-way ANOVA test at 5% confidence level. T, banana plants treated with ITBB B5-1 before infection with FOC4; CK1, banana plants treated with only LB medium before infection with FOC4; CK2, banana plants treated with LB medium to replace ITBB B5-1 and PDA medium to replace FOC4.

### Control effect of ITBB B5-1 against banana Fusarium wilt in the field

**Fig 6:**
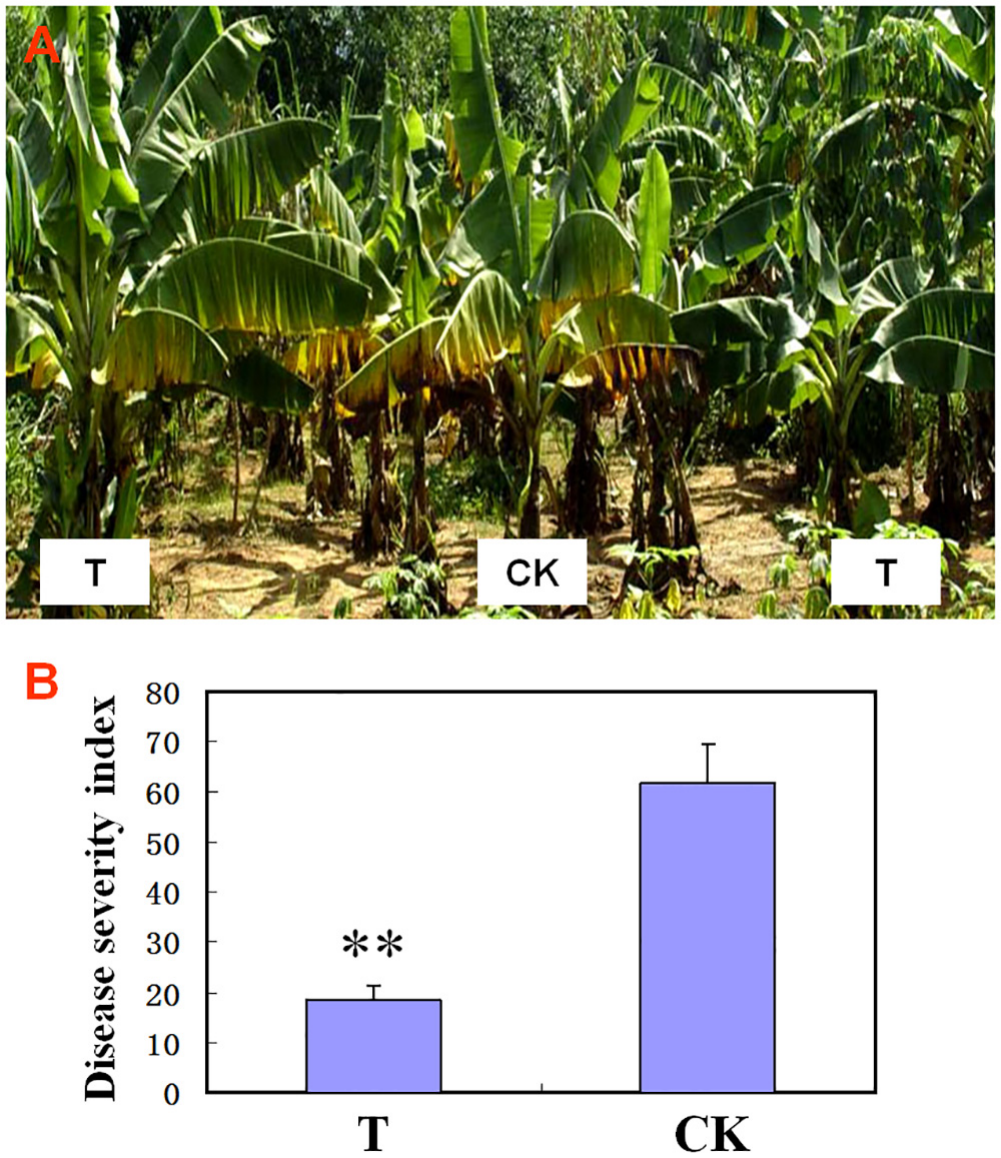
Inhibitory effect of ITBB B5-1 against Fusarium wilt of banana in the field. A, Plants after 6 months of growth in the field; B, Disease severity index of plants. CK, plants treated with only LB medium before infection with FOC4; T, plants treated with the ITBB B5-1 strain before infection with FOC4. “**” means significant difference between T and CK as analyzed with one-way ANOVA method at 1% confidence level.

Field experiments indicated that ITBB B5-1 significantly protected banana plants from developing Fusarium disease. The plants that were inoculated with ITBB B5-1 before infection with FOC4 had a disease severity index of only 18.3 after 6 months of infection, while the control plants treated with only LB medium before infection with FOC4 developed more severe Fusarium disease, with a disease severity index of 61.7. The control effect of ITBB B5-1 against Fusarium wilt disease in the field was approximately 70.0% (Fig 6).

## Discussion

The rubber tree is rich in endophytic microorganisms. The economic clones of the rubber tree reproduce by vegetative propagation known as budding, and the bark of the mature tree is regularly tapped. This agricultural process provides consistent wounds and pathways for microbes to invade and spread. We have isolated 18 endophytic fungal strains from the rubber tree [42], of which three strains demonstrated inhibition to the growth of the pathogenic fungus *Colletotrichum gloeosporioides* Penz. Sace, which causes the rubber tree anthracnose, and *Fusarium oxysporum* Cubense, which causes the banana Fusarium wilt. Another endophytic fungus, *Tritirachium sp*. ITBB2-1 exhibited salt resistance and optimum growth at a salt concentration of 600 mM NaCl [43]. Among the endophytic fungi isolated from the rubber tree, 80%–90% of them were *Ascomycota* species [42, 44], in which the sapwood presented a greater diversity than the leaves [44]. A novel algal genus *Heveochlorella* [45] and a novel fungal species *Trichoderma amazonicum* [46] were identified in the rubber tree.

The ITBB B5-1 strain was the first bacterial strain isolated from rubber tree, and was found to have antifungal activity. In this paper, we have demonstrated the potential of this strain in the biological control of banana Fusarium wilt caused by *Fusarium oxysporum* formae specialis *cubense* Race 4 (FOC4). This strain showed inhibitory effect on the mycelial growth and conidial formation of FOC4 (Fig 3), and the inhibition ratio to mycelial growth was quantified to be 95.4% (Fig 4). The antifungal effect was also demonstrated by green house test and field trials. Application of this strain reduced the disease severity index of nursery banana plants by 78.7% (Table 2), and protected the field plants from developing Fusarium disease by 70.0% (Fig 6).

Some *Serratia marcescens* strains have been shown to have potential in biological control of plant diseases. For example, a *Serratia* strain isolated from the rhizosphere of oilseed rape was demonstrated to have antifungal activity against different phytopathogenic fungi in vitro [47]. The strain SNB54 isolated from tobacco rhizosphere effectively suppressed black shank and root-knot diseases in tobacco in pot experiments [48]. A JPP1 strain isolated from peanut hulls was effective in inhibiting the mycelial growth of *Aspergillus parasiticus* and the production of aflatoxin [31]. Strains CFFSUR-B2, CFFSUR-B3, and CFFSUR-B4 inhibited the mycelial growth and conidial germination of the causal agent of fruit anthracnose *Colletotrichum gloeosporioides* [49]. However, most of the *Serratia* strains were isolated from rhizosphere or soil, and the antifungal activities were not demonstrated in field trials. Our strain ITBB B5-1 was an endophytic *Serratia* strain, and was demonstrated to have good protection effect of approximately 70.0% against banana Fusarium wilt in the field.

We have shown that chitinase and glucanase secreted by the ITBB B5-1 strain played a role in its antifungal activity (Fig 5). This antifungal mechanism was also suggested by Kalbe et al. based on the studies of some *Serratia* strains isolated from rhizosphere of oilseed rape [47]. Lytic enzymes, such as chitinases and β-1, 3 glucanases were common to *Serratia* strains [50–52]. However, the chitinase producing *S*. *marcescens* strain B2 alone did not inhibit fungal growth of *Fusarium oxysporum* f. sp. *Lycopersici*, but enhanced the biocontrol effect of an antibiotic producing bacterial strain against tomato Fusarium wilt [51]. Our strain ITBB B5-1 secreted both chitinase and glucanase, and significantly inhibited fungal growth of FOC4. Of course, the role of other products secreted by ITBB B5-1, such as prodigiosin, could not be ruled out of the antifungal mechanism.

## References

[1] Heslop-Harrison JS, Schwarzacher T (2007) Domestication, genomics and the future for banana. Ann Bot-London 100: 1073–1084. 10.1093/aob/mcm191 PMC2759213 17766312

[2] Jones DR (2009) Disease and pest constraints to banana production. ISHS Acta Horticulturae 828: 21–36.

[3] Ploetz RC (2000) Panama disease: a classic and destructive disease of banana. Plant Health Progr: 10.1094/PHP-2000-1204-1001-HM

[4] Ploetz RC (2006) Fusarium wilt of banana is caused by several pathogens referred to as *Fusarium oxysporum* f. sp. *cubense* . Phytopathology 96: 653–656. 10.1094/PHYTO-96-0653 18943184

[5] Fourie G, Steenkamp ET, Ploetz RC, Gordon TR, Viljoen A (2011) Current status of the taxonomic position of *Fusarium oxysporum* formae specialis *cubense* within the *Fusarium oxysporum* complex. Infect Genet Evol 11: 533–542. 10.1016/j.meegid.2011.01.012 21256980

[6] Ploetz RC, Pegg KG (1997) Fusarium wilt of banana and Wallace's line: Was the disease originally restricted to his Indo-Malayan region? Australas Plant Pathol 26: 239–249.

[7] CAB I (2005) *Fusarium oxysporum* f.sp. *cubense* (Panama disease of banana) Crop Protection Compendium. Wallingford, UK: CABI Publishing.

[8] Ploetz RC, Pegg KG (2000) Fusarium wilt In: Jones D. R., editor. Diseases of banana, abaca and enset. Wallingford, UK: CABI Publishing.

[9] Ploetz RC, Pegg KG (1999) Fusarium wilt In: Jones D. R., editor. Diseases of banana, abaca and enset. Wallingford, UK: CABI Publishing pp. 143–159.

[10] Hwang SC, Ko WH (2004) Cavendish banana cultivars resistant to Fusarium wilt acquired through somaclonal variation in Taiwan. Plant Dis 88: 580–588. 10.1094/PDIS.2004.88.6.580 30812575

[11] Lian J, Wang ZF, Cao LX, Tan HM, Inderbitzin P, Jiang Z, et al (2009) Artificial inoculation of banana tissue culture plantlets with indigenous endophytes originally derived from native banana plants. Biol Control 51: 427–434.

[12] Wang Z (2006) Advances in banana Fusarium wilt research and control. Plant Quar (in Chinese) 20: 198–200.

[13] Huang YH, Wang RC, Li CH, Zuo CW, Wei YR, Zhang L, et al (2012) Control of Fusarium wilt in banana with Chinese leek. Eur J Plant Pathol 134: 87–95. 23144534 10.1007/s10658-012-0024-3 PMC3491907

[14] Xu LB, Huang BZ, Wu YL, Huang YH, Dong T (2010) Production costs and benefits of banana production in wilt disease areas. Chin J Trop Agr 30: 38–44.

[15] Grimm D (2008) Plant genomics a bunch of trouble. Science 322: 1046–1047. 10.1126/science.322.5904.1046 19008426

[16] Saravanan T, Muthusamy M, Marimuthu T (2003) Development of integrated approach to manage the Fusarial wilt of banana. Crop Prot 22: 1117–1123.

[17] Smith MK, Hamill SD, Langdon PW, Giles JE, Doogan VJ, Pegg K (2006) Towards the development of a Cavendish banana resistant to race 4 of Fusarium wilt: gamma irradiation of micropropagated Dwarf Parfitt (Musa spp., AAA group, Cavendish subgroup). Aust J Exp Agr 46: 107–113.

[18] Nel B, Steinberg C, Labuschagne N, Viljoen A (2007) Evaluation of fungicides and sterilants for potential application in the management of Fusarium wilt of banana. Crop Prot 26: 697–705.

[19] Swarupa V, Ravishankar KV, Rekha A (2014) Plant defense response against *Fusarium oxysporum* and strategies to develop tolerant genotypes in banana. Planta 239: 735–751. 10.1007/s00425-013-2024-8 24420701

[20] Ghag SB, Shekhawat UK, Ganapathi TR (2014) Characterization of Fusarium wilt resistant somaclonal variants of banana cv. Rasthali by cDNA-RAPD. Mol Biol Rep 41: 7929–7935. 10.1007/s11033-014-3687-3 25160909

[21] Zhang H, Mallik A, Zeng RS (2013) Control of Panama disease of banana by rotating and intercropping with Chinese chive (*Allium tuberosum* Rottler): role of plant volatiles. J Chem Ecol 39: 243–252. 10.1007/s10886-013-0243-x 23355016

[22] Forsyth LM, Smith LJ, Aitken EA (2006) Identification and characterization of non-pathogenic *Fusarium oxysporum* capable of increasing and decreasing Fusarium wilt severity. Mycol Res 110: 929–935. 16891106 10.1016/j.mycres.2006.03.008

[23] Cao L, Qiu Z, You J, Tan H, Zhou S (2005) Isolation and characterization of endophytic streptomycete antagonists of Fusarium wilt pathogen from surface-sterilized banana roots. FEMS Microbiology Lett 247: 147–152. 10.1016/j.femsle.2005.05.006 15935565

[24] Paul JY, Becker DK, Dickman MB, Harding RM, Khanna HK, Dale JL (2011) Apoptosis-related genes confer resistance to Fusarium wilt in transgenic 'Lady Finger' bananas. Plant Biotechnol J 9: 1141–1148. 10.1111/j.1467-7652.2011.00639.x 21819535

[25] Yip MK, Lee SW, Su KC, Lin YH, Chen TY, Feng TY (2011) An easy and efficient protocol in the production of pflp transgenic banana against Fusarium wilt. Plant Biotechnol Rep 5: 245–254.

[26] Sambrook J, Fritsch EF, Maniatis T (1989) Molecular cloning: a laboratory manual, 2nd edition Cold Spring Harbor Laboratory. New York: Cold Spring Harbor.

[27] Wu W, Shi B (2008) Isolation and screening of thermostable lipase-producing bacterium FS1403 and analysis of its 16S rDNA gene sequence. Pharm Biotechnol 15: 6–10.

[28] Chenna R, Sugawara H, Koike T, Lopez R, Gibson TJ, Higgins DG, et al (2003) Multiple sequence alignment with the Clustal series of programs. Nucl Acids Res 31: 3497–3500. 12824352 10.1093/nar/gkg500 PMC168907

[29] Tamura K, Dudley J, Nei M, Kumar S (2007) MEGA4: Molecular Evolutionary Genetics Analysis (MEGA) software version 4.0. Mol Biol Evol 24: 1596–1599. 17488738 10.1093/molbev/msm092

[30] Rzhetsky A, Nei M (1992) A simple method for estimating and testing minimum evolution trees. Mol Biol Evol 9: 945–967.

[31] Wang K, Yan P, Cao L, Ding Q, Shao C, Zhao TF (2013) Potential of chitinolytic *Serratia marcescens* strain JPP1 for biological control of *Aspergillus parasiticus* and Aflatoxin. BioMed Res Int 2013 : Article ID 397142. 10.1155/2013/397142 PMC3705848 23865052

[32] Yabe K, Nakamura H, Ando Y, Terakado N, Nakajima H, Hamasaki T (1988) Isolation and characterization of *Aspergillus parasiticus* mutants with impaired aflatoxin production by a novel tip culture method. Appl Environ Microbial 54: 2096–2100. 10.1128/aem.54.8.2096-2100.1988 PMC202809 3178213

[33] Monreal J, Reese ET (1969) The chitinase of *Serratia marcescens* . Can J Microbiol 15: 689–696. 4894282 10.1139/m69-122

[34] Fink W, Liefland M, Mendgen K (1988) Chitinases and, β-1, 3-glucanases in the apoplastic compartment of oat leaves (*Avena sativa* L.). Plant Physiol 88: 270–275. 16666294 10.1104/pp.88.2.270 PMC1055567

[35] Orjeda G (1998) Evaluation of Musa germplasm for resistance to sigatoka disease and Fusarium wilt In: I. P. G. R. Institute, editor. INIBAP Technical Guidelines III. Rome, Italy.

[36] Williamson NR, Fineran PC, Leeper FJ, Salmond GP (2006) The biosynthesis and regulation of bacterial prodiginines. Nat Rev Microbiol 4: 887–899. 17109029 10.1038/nrmicro1531

[37] Xu F, Xia S, Yang Q (2011) Strategy for obtaining inexpensive prodigiosin production by *Serratia marcescen*. 3rd International Conference on Chemical, Biological and Environmental Engineering.

[38] Kalivoda EJ, Brothers KM, Stella NA, Schmitt MJ, Shanks RM (2014) Bacterial cyclic AMP-phosphodiesterase activity coordinates biofilm formation. PloS One 8: e71267. 10.1371/journal.pone.0071267 PMC3726613 23923059

[39] Ajithkumar B, Ajithkumar VP, Iriye R, Doi Y, Sakai T (2003) Spore-forming *Serratia marcescens subsp*. *sakuensis subsp*. *nov*., isolated from a domestic wastewater treatment tank. Int J Syst Evol Microbiol 53: 253–258. 12656181 10.1099/ijs.0.02158-0

[40] Bruton BD, Fletcher J, Pair SD, Shaw M, Sittertz-Bhatkar H (1998) Association of a phloem-limited bacterium with yellow vine disease in cucurbits. Plant Dis 82: 512–520. 10.1094/PDIS.1998.82.5.512 30856981

[41] Yi S, Wang W, Bai F, Zhu J, Li J, Li X, et al (2014) Antimicrobial effect and membrane-active mechanism of tea polyphenols against *Serratia marcescens* . World J Microb Biot 30: 451–460. 10.1007/s11274-013-1464-4 23979827

[42] Zheng P, He J, Chang K, Zhang S, Tan D, Sun X, et al (2009) Isolation and identification of endophytic fungus from rubber tree and their antagonism to plant pathogens. Chin J Trop Crops 30: 832–837.

[43] Zheng P, Tan D, Sun X, Zhang J (2009) Morphology and phylogenetic position of an endophytic fungus ITBB2-1 from rubber tree. Chin J Trop Crops 30: 314–319.

[44] Gazis R, Chaverri P (2010) Diversity of fungal endophytes in leaves and stems of wild rubber trees (*Hevea brasiliensis*) in Peru. Fung Ecol 3: 240–254.

[45] Zhang J, Huss VAR, Sun X, Chang K, Pang D (2008) Morphology and phylogenetic position of a trebouxiophycean green alga (Chlorophyta) growing on the rubber tree, *Hevea brasiliensis*, with the description of a new genus and species. Eur J Phycol 43: 185–193.

[46] Chaverri P, Romina O, Gazis RO, Samuels GJ (2011) *Trichoderma amazonicum*, a new endophytic species on *Hevea brasiliensis* and *H*. *guianensis* from the Amazon basin. Mycologia 103: 139–151. 10.3852/10-078 20943534

[47] Kalbe C, Marten P, Berg G (1996) Strains of the genus *Serratia* as beneficial rhizobacteria of oilseed rape with antifungal properties. Microbiol Res 151: 433–439. 9022304 10.1016/S0944-5013(96)80014-0

[48] Huang Y, Ma L, Fang DH, Xi JQ, Zhu ML, Mo MH, et al (2015) Isolation and characterisation of rhizosphere bacteria active against *Meloidogyne incognita*, *Phytophthora nicotianae* and the root knot-black shank complex in tobacco. Pest Manag Sci 71: 415–422. 10.1002/ps.3820 24799254

[49] Gutierrez-Roman MI, Holguin-Melendez F, Bello-Mendoza R, Guillen-Navarro K, Dunn MF, Huerta-Palacios G (2012) Production of prodigiosin and chitinases by tropical *Serratia marcescens* strains with potential to control plant pathogens. World J Microb Biot 28: 145–153. 10.1007/s11274-011-0803-6 22806790

[50] Wang K, Yan PS, Cao LX (2014) Chitinase from a novel strain of *Serratia marcescens* JPP1 for biocontrol of aflatoxin: molecular characterization and production optimization using response surface methodology. Biomed Res Int 2014: 482623 10.1155/2014/482623 24812619 PMC4000942

[51] Someya N, Tsuchiya K, Yoshida T, Noguchi MT, Akutsu K, Sawada H (2007) Co-inoculation of an antibiotic-producing bacterium and a lytic enzyme-producing bacterium for the biocontrol of tomato wilt caused by *Fusarium oxysporum* f. sp. *lycopersici* . Biocontrol Sci 12: 1–6. 17408002 10.4265/bio.12.1

[52] Frankowski J, Lorito M, Scala F, Schmid R, Berg G, Bahl H (2001) Purification and properties of two chitinolytic enzymes of *Serratia plymuthica* HRO-C48. Arch Microbiol 176: 421–426. 11734885 10.1007/s002030100347

